# Diagnostic Accuracy of Dermoscopic Characteristics in Cutaneous Squamous Cell Carcinoma: A Retrospective Analysis

**DOI:** 10.1155/jskc/9944605

**Published:** 2025-10-15

**Authors:** Jia-Li Zhang, Guan-Zhi Chen, Da Wang, Xiao-Ou Lu, Wei-Wei Fu, Chang-Qing Shi, Hong-Quan Chen

**Affiliations:** ^1^Department of Dermatology, The Affiliated Hospital of Qingdao University, Pingdu, Qingdao, Shandong 266000, China; ^2^Department of Dermatology, The Affiliated Hospital of Qingdao University, Qingdao, Shandong 266005, China; ^3^Department of Dermatology, Yantai Yuhuangding Hospital Affiliated to Qingdao University Medical College, Yantai, Shandong 264000, China

**Keywords:** cutaneous squamous cell carcinoma, dermoscopy, differential diagnosis

## Abstract

**Objective:**

This study aims to evaluate the diagnostic efficacy of dermoscopy for cutaneous squamous cell carcinoma (cSCC).

**Methods:**

A retrospective analysis was conducted on skin lesions diagnosed as cSCC via histopathology at the Department of Dermatology, Pingdu District, The Affiliated Hospital of Qingdao University, between January 2021 and August 2024 (case group). Lesions suspected of cSCC but ultimately diagnosed as other conditions served as the control group. The study compared disease progression and dermoscopic features between the two groups through chi-square test and logistic regression analysis, using histopathological results as the diagnostic reference standard.

**Results:**

The case group consisted of 41 lesions, while the control group comprised 18 lesions diagnosed with conditions including actinic keratosis, basal cell carcinoma, and keratoacanthoma. The clinical misdiagnosis rate was approximately 30.5%. Notably, a disease duration of less than 2 years (28/41, 68.29%), the presence of white circles (21/41, 51.22%) and blood spots (35/41, 85.37%), and a total of ≥ 5 dermoscopic features (19/41, 46.34%) were significantly more prevalent in the case group compared to the control group (both *p* < 0.05). Among these, a disease duration of less than 2 years, along with the presence of white circles and blood spots, demonstrated statistical significance in differentiating cSCC from other conditions (both *p* < 0.05).

**Conclusions:**

For patients with clinically suspected cSCC, a disease duration of less than 2 years and dermoscopic observation of white circles and blood spots provide substantial diagnostic value.

## 1. Introduction

In recent years, the emergence of noninvasive imaging technology has provided clinicians with swifter, more accessible diagnostic methods that eliminate the need for invasive procedures. This advancement is crucial in dermatological diagnosis, offering vital insights for thorough preoperative evaluations of patients with skin tumors and enabling the development of personalized, comprehensive treatment plans. Dermoscopy, a noninvasive examination technique, allows visualization of skin structures from the epidermis to the dermal papillary layer. By identifying pigment patterns, vascular features, and other local lesion characteristics, dermoscopy assists in distinguishing between benign and malignant conditions, serving as a valuable adjunct for assessing clinically visible lesions [1]. Numerous studies have demonstrated dermoscopy's effectiveness in improving melanoma diagnosis [2], and there is a growing body of research exploring its application in nonmelanoma skin cancers. cSCC, the second most common nonmelanoma skin cancer after basal cell carcinoma [3], is known for its high clinical misdiagnosis rate and is the deadliest within its category [4]. Primarily affecting elderly men, it typically arises in sun-exposed areas. The etiology of cSCC is multifactorial, involving factors such as ultraviolet radiation exposure, carcinogen contact, preexisting skin conditions, scars, trauma, and immunosuppression. Clinically, it may present as expanding dark red plaques, nodules, or ulcers resembling cauliflower or papillomas. Initial symptoms are localized, but the disease can progress to local invasion, regional lymph node metastasis, and systemic involvement in advanced stages [5]. A swift and accurate diagnosis, along with prompt and effective intervention and treatment, is crucial for optimizing patient outcomes. Given the cSCC's heterogeneous clinical presentations, it is essential to distinguish it from conditions such as actinic keratosis, basal cell carcinoma, seborrheic keratosis, and skin ulcers.

Researchers have delineated distinct dermoscopic features associated with different grades of differentiation of cSCC, and the research indicates that poorly differentiated cSCC typically exhibits a polymorphic vascular pattern on dermoscopy, featuring numerous linear, loop-shaped, and coiled vessels, as well as occasional white, unstructured areas. Moderately differentiated cSCC is more often characterized by peripheral loop-shaped vessels and diffuse yellow to light brown unstructured regions. Well-differentiated cSCC, as seen on dermoscopy, presents with central yellowish-white keratin, scales, a polymorphic vascular pattern, pearl-like structures, white annuli, and blood crusts [6–8]. These findings hold potential value for the early diagnosis of cSCC. Furthermore, dermoscopy can accurately distinguish Bowen's disease, marked by clustered glomerular vessels, from keratoacanthoma and invasive cSCC [9]. However, despite these advancements, research on the differential diagnosis of cSCC based on dermoscopic features remains limited, and the influence of disease duration and the total number of dermoscopic features on differential diagnosis has not been thoroughly explored. This study incorporates disease duration and the total number of dermoscopic features to investigate the application value of dermoscopy in the differential diagnosis of cSCC, aiming to enhance clinicians' noninvasive diagnostic capabilities and improve the accuracy of clinical diagnosis.

## 2. Materials and Methods

### 2.1. Instrumentation

All dermoscopic images were captured using the Nanjing Beining BN-PFMF-8001 dermoscopy image analysis system at a 35x magnification. This noncontact, polarized light electronic dermoscope functions effectively without requiring immersion fluids.

### 2.2. Dermoscopic Features of cSCC

Based on relevant literature [8, 9] and our clinical expertise, we identified the following dermoscopic features characteristic of cSCC: (1) central keratin mass; (2) scales; (3) polymorphous vasculature, including at least two of the following: ① hairpin-irregular vessels, ② linear-irregular vessels, and ③ dotted and glomerular vessels; (4) keratin pearls; (5) white circles; (6) white, structureless areas; (7) ulceration; (8) blood spots; (9) brown to gray globules/dots; and (10) rosettes.

### 2.3. Study Population and Evaluation Methods

This retrospective cross-sectional study was conducted on the 59 dermoscopic images of patients clinically suspected of having cSCC at the Dermatology Department of Pingdu Branch, Qingdao University Affiliated Hospital, from January 2021 to August 2024. The study was approved by the Medical Ethics Committee of Qingdao University Affiliated Hospital (QYFY WZLL 28718). Informed consent was obtained from all patients for the publication of case details and use of images. We assembled a case group consisting of patients diagnosed with cSCC who underwent comprehensive dermoscopy and received pathological confirmation. For the control group, we included patients clinically suspected of having cSCC but confirmed by pathology to have other skin diseases. The images from both groups, totaling 59, were randomly ordered. Two dermatologists, trained in dermoscopy at the National Telemedicine and Internet Medicine Center and certified in skin imaging, retrospectively reviewed these dermoscopic images. They reached a consensus on the presence or absence of specific dermoscopic features for each lesion. In the analysis, we first compared the disease duration and dermoscopic feature differences between the two groups; Subsequently, we selected features with statistically significant differences and assessed their diagnostic utility for cSCC.

### 2.4. Statistical Methods

Data were recorded in an Excel spreadsheet and subsequently analyzed using SPSS software, Version 25.0. We employed the chi-square test to compare disease duration and dermoscopic features between the two groups. In cases where the assumptions for the chi-square test were not satisfied, we utilized either the continuity correction or Fisher's exact test. A *p* value of less than 0.05 was deemed statistically significant. For features showing statistical significance, logistic regression analysis was conducted to calculate odds ratios, *p* values, and 95% confidence intervals, with statistical significance again defined as *p* < 0.05.

## 3. Results

### 3.1. General Information

The case group consisted of 41 skin lesions from 36 patients, including 11 males (30.6%) and 25 females (69.4%), with ages ranging from 50 to 94 years and a mean age of 75.2 years. The disease duration varied from 2 weeks to 6 years. Among these patients, 3 had multiple cSCCs, and 1 had a history of lymphoma. Lesions were located on the cheeks (28 cases), temples (5 cases), nose (3 cases), scalp (1 cases), neck (1 cases), dorsum of the left hand (1 case), left waist (1 case), and inner aspect of the right thigh (1 case). The lesions presented as 23 elevated nodular lesions and 18 plaques with erosions, ulcers, or crusts. Notably, 5 cases developed from multiple actinic keratoses, 2 from keratoacanthomas, and 1 was associated with a chronic ulcer.

The control group included 18 skin lesions from 16 patients, comprising 4 males (25%) and 12 females (75%), with ages spanning from 58 to 98 years and an average age of 76.9 years. The duration of these lesions ranged from 2 months to 8 years. Lesions were anatomically distributed as follows: cheeks (10 cases), temples (2 cases), nose (2 cases), forehead (1 case), periorbital area (1 case), chin (1 case), and right hip (1 case). The identified skin conditions included solar keratosis (7 cases), basal cell carcinoma (6 cases), keratoacanthoma (4 cases), and one proliferating trichilemmal cyst (1 case).

### 3.2. Frequency of Appearance of Each Skin Lesion Feature

**Figure 1:**
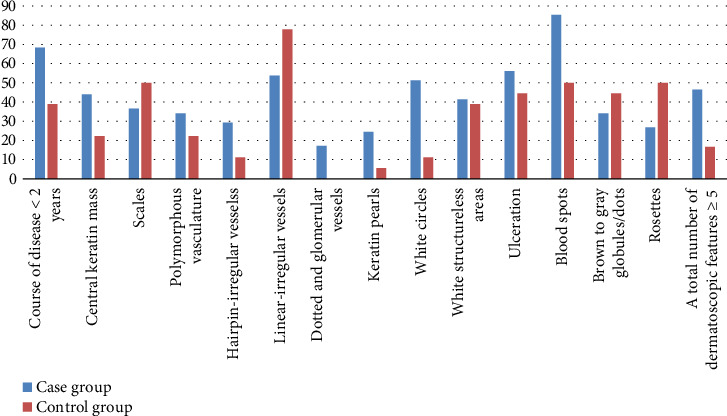
Frequency of dermoscopic features and course of disease (%).

**Figure 2:**
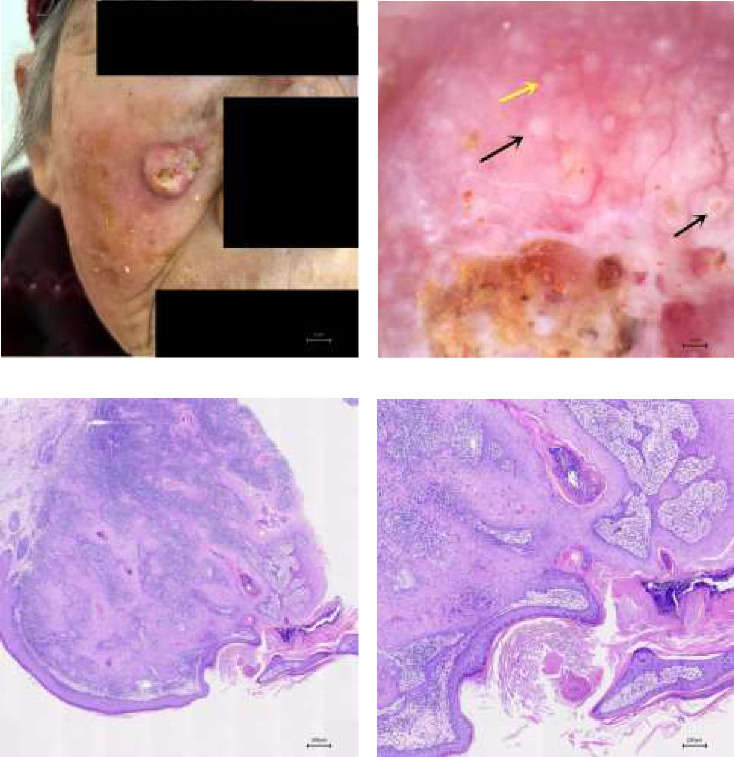
(a) Moderately differentiated cSCC, a single tubercle 2 cm in diameter on the right cheek. (b) Dermoscopy displays a dark red nodule, central yellow–brown keratin mass, white unstructured areas, irregular linear vessels, white circles (black arrow), and rosettes (yellow arrow) (× 35). (c) Histopathology: moderately differentiated cSCC (× 40). (d) Histopathology: moderately differentiated cSCC (× 100).

**Figure 3:**
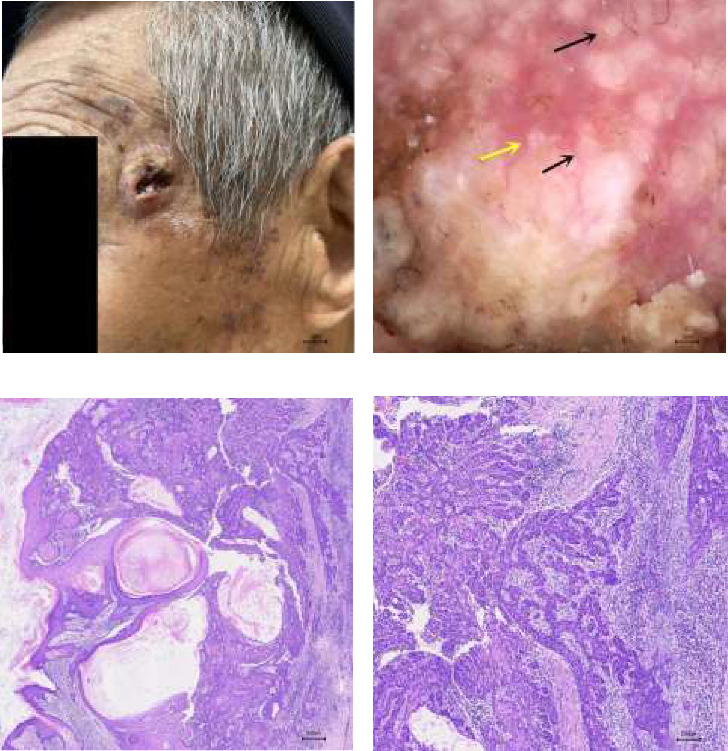
(a) Moderately differentiated cSCC, a crater-like nodules and ulceration in the left temporal area, about 2.2 cm in diameter. (b) The central ulcer and blood scab can be observed under dermoscopy. White unstructured area, irregular linear blood vessels, white circles (black arrow), and rosettes (yellow arrow) can be seen around it (× 35). (c) Histopathology: moderately differentiated cSCC (× 40). (d) Histopathology: moderately differentiated cSCC (× 100).

**Figure 4:**
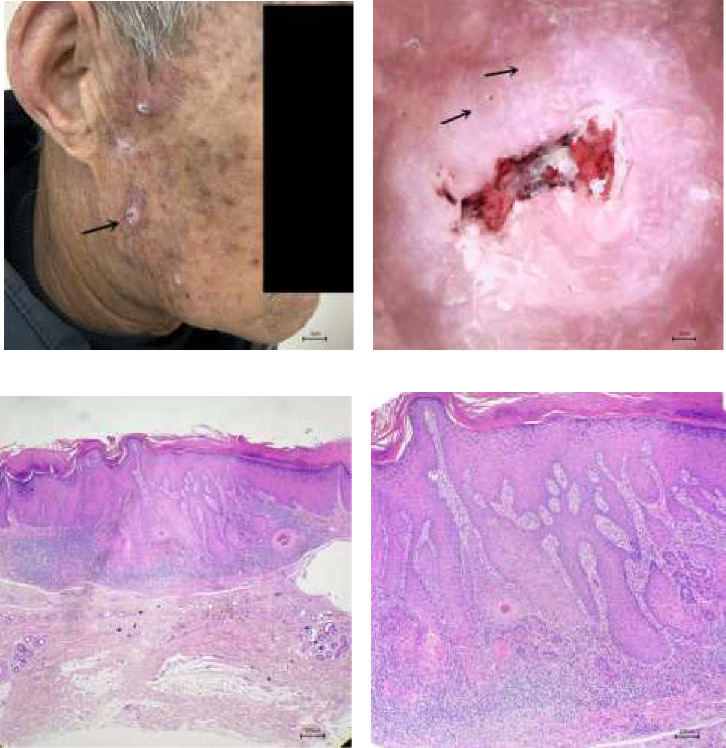
(a) Well-differentiated cSCC, multiple nodules in the right temporal area, and about 0.6–1 cm in diameter. (b) (Corresponding to the lesion indicated by the black arrow) Dermoscopic pink–white papules with a central brown keratin mass and blood scab, as well as surrounding keratin pearls (black arrow) (× 35). (c) Histopathology: well-differentiated cSCC (× 40). (d) Histopathology: well-differentiated cSCC (× 100).

A disease duration of less than 2 years, the presence of white circles and blood spots, and a total of five or more dermatoscopic features were significantly associated with the differential diagnosis of cSCC (all *p* < 0.05). These features are detailed in Table 1 and illustrated in Figure 1. Figures 2, 3, and 4 showcase the principal clinical, dermoscopic, and histological features.

### 3.3. The Evaluation of the Diagnostic Value of Disease Duration and the Dermoscopic Features for cSCC

Key factors identified for the differential diagnosis of cSCC include a disease duration of less than 2 years, the presence of white circles and blood scabs on dermatoscopy, and a total of five or more dermatoscopic features. Binary logistic regression analysis confirmed that these parameters—particularly a disease duration of less than 2 years, the presence of white circles, and blood scabs observed dermoscopically—are statistically significant indicators for cSCC diagnosis (all *p* < 0.05), as detailed in Table 2.

## 4. Discussion

Dermatopathology remains the gold standard for diagnosing skin neoplasms. However, with the progress in skin imaging technologies, noninvasive optical techniques such as dermoscopy have proven effective in enhancing the accuracy of clinical diagnoses and uncovering specific internal characteristics of skin tumors [10]. Dermoscopy, a convenient and noninvasive adjunctive tool, stands out as one of the most advanced and thoroughly researched diagnostic methods in dermatologic imaging, widely employed in clinical practice to aid in skin condition diagnosis. Nonmelanoma skin cancer, a collective term for skin malignancies, encompasses common types such as basal cell carcinoma and cSCC, along with the rarer Merkel cell carcinoma [11]. Research indicates that the clinical and pathological features of nonmelanoma skin cancer differ between Caucasian and Asian populations [12, 13], underscoring the necessity of exploring the diagnostic utility of dermoscopic features among Asian individuals, including the Chinese. cSCC, a major form of nonmelanoma skin cancer, accounts for roughly 20% of malignant skin neoplasms and, excluding melanoma, is responsible for about 75% of skin cancer-related mortalities [14]. Early and accurate diagnosis and treatment of cSCC are crucial to prevent tumor progression and improve patient outcomes.

In this study, the average age of cSCC patients was 75.2 years, consistent with the higher age incidence reported in the existing literature, confirming age as a notable risk factor for cSCC. Notably, our cohort had a lower proportion of male patients compared to previous research that indicated a male predominance [15–17]. This discrepancy may stem from women in this region having greater aesthetic and social concerns, prompting more proactive engagement in diagnostic and treatment processes. Consequently, a higher number of women were willing to undergo dermoscopy and histopathological evaluation, leading to their inclusion in this study. The cheek was the most common site for cSCC (28/41, 68.3%), followed by other sun-exposed areas such as the temple and nose. This distribution aligns with prior findings and suggests a connection to chronic ultraviolet exposure, highlighting the importance of targeted preventive skin examinations in these high-risk regions. We observed multiple cSCC occurrences in 8.3% of cases, suggesting potential immune dysfunction, DNA repair anomalies, or other predisposing factors that elevate recurrence risk and necessitate more rigorous patient follow-up. In this cohort, cSCC developed from multiple actinic keratoses in 4 cases, from keratoacanthoma in 2, and from chronic ulcers in 1, underscoring the need for heightened vigilance and regular monitoring in patients with these conditions. The control group included conditions such as actinic keratosis, basal cell carcinoma, and keratoacanthoma, which can be clinically confused with cSCC due to similar lesion presentations, potentially leading to misdiagnosis. Our case group comprised 41 lesions, while the control group had 18, resulting in a clinical misdiagnosis rate of approximately 30.5%. According to relevant reports, the average misdiagnosis rate of skin malignant tumors in China's top three hospitals is about 30%, and in primary medical institutions, which serve the largest patient base, the rate can reach as high as 70% [18]. Therefore, employing convenient, noninvasive adjunctive tests to improve diagnostic accuracy for malignant skin neoplasms holds significant clinical importance.

Under dermoscopy, features such as white circles (21/41, 51.22%) and blood spots (35/41, 85.37%) have been linked to differentiating cSCC, both showing statistical significance (*p* < 0.05). In a study by Dimitrios Sgouros et al. [19], white circles, cornified material, blood spots, and white structureless areas were identified as dermoscopic indicators distinguishing cSCC and actinic keratosis from other nonpigmented skin disorders. Our study observed a slightly lower incidence of white circles (51.22%) compared to Rosendahl's findings (60%), but a higher occurrence of blood spots (85.37% vs. 41.7%). These differences may arise from variations in lesion stages, locations, and sample sizes across studies. Dermoscopically, white circles manifest as bright, white annuli encircling dilated follicular infundibulum and correspond histologically to thickened spinous and granular epidermal layers at the center of the infundibular opening [20]. This likely reflects the proliferative and keratinizing nature of cSCC cells, appearing as ring-like or target-shaped features, also seen in actinic keratosis. cSCC can be misdiagnosed as actinic keratosis since both commonly occur in sun-exposed areas and clinically present as dark red plaques with yellow–white scaling. Notably, 0.1%–16% of actinic keratosis cases may progress to cSCC, with a significantly higher transformation rate in patients with multiple lesions compared to a single one [21]. Li et al. [22] propose that a significant rise in vascularity and keratin structures observed under dermoscopy signifies the progression from actinic keratosis to invasive cSCC. Similarly, Ertop Doğan et al. [23] and other researchers caution that the concurrent presence of white structureless areas and ulcers during dermoscopic examination may indicate a transition from actinic keratosis to cSCC. To gain a clearer understanding of these dermoscopic features, larger-scale sample analyses are essential. Blood spots, another notable dermoscopic marker, are associated with cSCC characteristics such as vascular proliferation and fragility. Moreover, this study reveals that the emergence of lesions within a 2-year timeframe holds statistical significance in differentiating cSCC. Although this finding is novel and not previously documented, potential recall biases or inaccuracies in the existing literature could influence the results. Nevertheless, this discovery offers promising prospects for enhancing the diagnostic precision of cSCC.

It should be recognized that this study is constrained by a limited sample size, with the patient cohort drawn exclusively from the Pingdu area of Qingdao, which restricts the generalizability of the findings. Additionally, there is a notable verification bias, as cases were confirmed using a single gold standard, while controls relied on alternative verification methods or reference tests. Moreover, the use of a polarized light skin microscope in this study presents another limitation. While nonpolarized microscopes offer superior image recognition for keratin-rich substances, the polarized light microscope employed here may have impacted the accurate identification of such features. To address these limitations, future research will aim to enlarge the scope of the study, encompassing a wider dermoscopic range and a larger sample size, to validate the diagnostic significance of these dermoscopic features more robustly. Concurrently, it is of utmost importance to optimize dermoscopic criteria, which can be achieved by better capturing the representation of dermoscopic features in lesions, incorporating nonpolarized dermoscopy techniques, and adding additional vascular pattern types. Such measures are designed to improve the diagnostic precision of dermoscopy for cSCC. Finally, while dermoscopy is a viable tool for the early screening, differential diagnosis, and dynamic monitoring of cSCC, skin histopathology remains the gold standard for definitive diagnosis. Therefore, timely histopathological evaluation of suspicious lesions to exclude malignancy is essential. The integration of dermoscopy is anticipated to contribute to earlier and more accurate diagnoses.

In conclusion, dermoscopy holds substantial clinical value for diagnosing and differentiating cSCC. As scientific data evolve, this technique is poised to become an indispensable adjunct in the routine diagnostic and therapeutic toolkit of clinical practice.

## Figures and Tables

**Table 1:** Frequency of dermoscopic features and disease duration in cSCC and non-cSCC Groups (*n* [%]).

	Case group (*n* = 41)	Control group (*n* = 18)	*χ* ^2^	*p*
Course of disease < 2 years	28 (68.29)	7 (38.89)	4.482	0.034
Dermatoscopic features				
Central keratin mass	18 (43.90)	4 (22.22)	2.541	0.113
Scales	15 (36.59)	9 (50)	0.933	0.334
Polymorphous vasculature	14 (34.15)	4 (22.22)	0.839	0.36
Hairpin-irregular vessels	12 (29.27)	2 (11.11)	1.386	0.239
Linear-irregular vessels	22 (53.66)	14 (77.78)	3.059	0.08
Dotted and glomerular vessels	7 (17.07)	0	2.045	0.153
Keratin pearls	10 (24.39)	1 (5.56)	1.815	0.178
White circles	21 (51.22)	2 (11.11)	8.46	0.004
White structureless areas	17 (41.46)	7 (38.89)	0.034	0.853
Ulceration	23 (56.10)	8 (44.44)	0.681	0.409
Blood spots	35 (85.37)	9 (50)	6.492	0.011
Brown to gray globules/dots	14 (34.15)	8 (44.44)	0.567	0.451
Rosettes	11 (26.83)	9 (50)	2.997	0.083
A total number of dermatoscopic features^∗^ ≥ 5	19 (46.34)	3 (16.67)	4.71	0.03

^∗^The total number of skin features other than blue–gray granules and rose petal sign appearing in each lesion.

**Table 2:** Logistic regression analysis results.

	B	Wald	*p*	OR	95% CI
Course of disease < 2 years	1.773	5.197	0.023	5.888	1.282–27.040
White circles	2.818	4.727	0.030	16.737	1.320–212.205
Blood spots	2.433	6.364	0.012	11.398	1.721–75.491
A total number of dermatoscopic features ≥ 5	−0.291	1.558	0.852	0.748	0.035–15.845

## Data Availability

The data that support the findings of this study are available on request from the corresponding author (Dr. Hong-Quan Chen, E-mail: chenhongquan0811@163.com). The data are not publicly available due to privacy or ethical restrictions.
